# Fast Imaging Technique for fMRI: Consecutive Multishot Echo Planar Imaging Accelerated with GRAPPA Technique

**DOI:** 10.1155/2015/394213

**Published:** 2015-08-27

**Authors:** Daehun Kang, Yul-Wan Sung, Chang-Ki Kang

**Affiliations:** ^1^Kansei Fukushi Research Institute, Tohoku Fukushi University, Sendai 989-3201, Japan; ^2^Graduate School of Information Science, Tohoku University, Sendai 980-8579, Japan; ^3^Neuroscience Research Institute and Department of Radiological Science, Gachon University, 1198 Kuwol-dong, Namdong-gu, Incheon 405-760, Republic of Korea

## Abstract

This study was to evaluate the proposed consecutive multishot echo planar imaging (cmsEPI) combined with a parallel imaging technique in terms of signal-to-noise ratio (SNR) and acceleration for a functional imaging study. We developed cmsEPI sequence using both consecutively acquired multishot EPI segments and variable flip angles to minimize the delay between segments and to maximize the SNR, respectively. We also combined cmsEPI with the generalized autocalibrating partially parallel acquisitions (GRAPPA) method. Temporal SNRs were measured at different acceleration factors and number of segments for functional sensitivity evaluation. We also examined the geometric distortions, which inherently occurred in EPI sequence. The practical acceleration factors, *R* = 2 or *R* = 3, of the proposed technique improved the temporal SNR by maximally 18% in phantom test and by averagely 8.2% in in vivo experiment, compared to cmsEPI without parallel imaging. The data collection time was decreased in inverse proportion to the acceleration factor as well. The improved temporal SNR resulted in better statistical power when evaluated on the functional response of the brain. In this study, we demonstrated that the combination of cmsEPI with the parallel imaging technique could provide the improved functional sensitivity for functional imaging study, compensating for the lower SNR by cmsEPI.

## 1. Introduction

In functional magnetic resonance imaging (fMRI) studies, echo planar imaging (EPI) technique has been widely used for investigating brain functions in which the signal is based on blood-oxygen-level-dependent (BOLD) contrast, reflecting the relationship between neuronal activity and concentration of deoxyhemoglobin in a blood vessel [1, 2]. Since EPI is one of the fastest imaging techniques, it has been suitable for observing functional dynamic changes of the brain. For high-resolution functional imaging, a segmented EPI, that is, multishot EPI (msEPI), has been employed as an alternative to a typical single-shot EPI (ssEPI) [2–5], because ssEPI image showed severe geometrical distortion and signal loss caused by accumulated magnetic susceptibility or field inhomogeneity. Also, the effective spatial resolution became worse by T_2_^*∗*^ filter effect of a tissue, as a readout period in ssEPI increases [3, 6, 7].

In the previous study [4], the authors suggested msEPI to be performed by the acquisition of all the segments in a single slice before continuing on to the next slices in turn. The study demonstrated that optimum contrast sensitivity in BOLD-based fMRI experiments using msEPI could be achieved by using the short repetition time (TR) values between segments and the long echo train length. The short TR between segments was achievable with minimized intersegment delay. The other studies suggested variable flip angles (VFA) to maximize signal-to-noise ratio (SNR) for a short duration of a segment, rather than the Ernst angle [5, 8]. Thus, both the minimum intersegment delay and VFA were employed to optimize msEPI for functional imaging. This technique was named as interleaved or snapshot EPI in the previous studies [5, 8]. To avoid confusing with other multishot EPI techniques, we called it as consecutive multishot EPI (cmsEPI).

In the meantime, the advance of RF multicoil arrays and their encoding capability has made the parallel imaging acquisition possible, which was associated with the significant scan time reduction in many clinical applications. Many parallel imaging reconstruction methods such as sensitivity encoding (SENSE) [9], simultaneous acquisition of spatial harmonics (SMASH) [10], and generalized autocalibrating partially parallel acquisitions (GRAPPA) [11] have been suggested. However, they also come with a nonuniform noise enhancement and then with a nonuniform loss in SNR compared to nonaccelerated images as presented in the previous studies [9, 12, 13]. Nevertheless, the utilization of the parallel imaging acquisitions became essential due to the enhancement of imaging speed and sensitivity, especially at high field MRI above 3T [14].

The combined technique, however, of cmsEPI with a parallel imaging has not been reported in ultrahigh field 7T MRI. In this study, therefore, we investigated cmsEPI with GRAPPA technique to improve SNR and evaluated the functional sensitivity of the proposed technique.

## 2. Materials and Methods

### 2.1. Sequence Design

**Figure 1:**
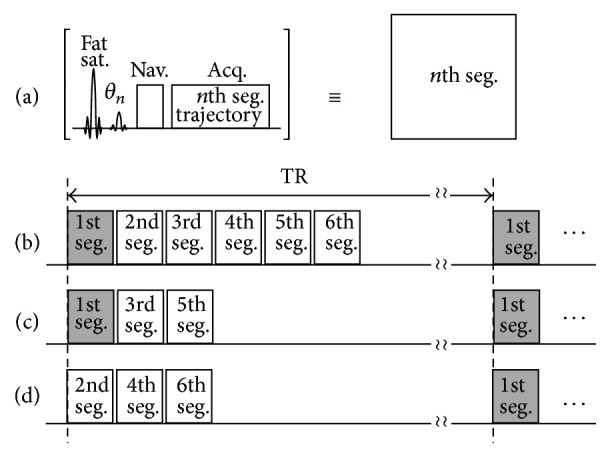
Simplified pulse sequence diagram. (a) The definition of a segment in multishot EPI, which consists of a fat-saturating RF pulse, a slice-selective RF pulse, navigators, and a data acquisition. Timing diagrams of (b) cmsEPI for 6 segments and (c) accelerated cmsEPI with reduction factor of *R* = 2 for 6 segments. For the reference data of *R* = 2, all six segments need to be acquired but in two sets: one includes 1st, 3rd, and 5th segments and the other 2nd, 4th, and 6th segments as described in (d). Fat Sat.: fat saturation; FA: flip angle; Nav.: navigation echo; Seg.: segment; TR: repetition time or time for acquisition of one slice.

Each segment of cmsEPI pulse sequence consisted of a fat-saturating RF pulse, a slice-selective RF pulse, navigators, and a data acquisition as plotted in Figure 1(a). The minimized interval between the segments required VFA for the slice-selective RF pulses, which allowed the segments to have an equivalent transverse magnetization theoretically. A typical Cartesian k-space was filled with k-space trajectories as many as the number of segments without overlapping. The relative size of a blip along a phase-encoding direction of each segment was also the same as the number of segments.

For the accelerated acquisition, the number of segments to be measured was reduced by the factor of 1/*R*, in which *R* denoted the reduction or acceleration factor. The corresponding modified VFA were also adjusted to the reduced number of segments, which was defined as the following:

(1) $$\begin{array}{c} \theta_{n}=\sin^{-1}\left(\frac{1}{\sqrt{N/R-n+1}}\right), \end{array}$$

where *θ*_*n*_, *N*, and *n* denote the *n*th flip angle, the number of segments, and an integer in range between 1 and *N*/*R*, respectively.

For 6 segments, for example, a sequence of flip angles was 24°, 26°, 30°, 35°, 45°, and 90° in order, as shown in Figure 1(b). In a reduction factor of 2 (*R* = 2), only three flip angles were required, that is, 35°, 45°, and 90°, in order. In a k-space with a reduction factor of 2, three segments were chosen to make a trajectory with a constant interval along phase-encoding direction. Two measurements were conducted for the reference lines, each of which had 3 segments (1st, 3rd, and 5th segments or 2nd, 4th, and 6th segments) as described in Figures 1(c) and 1(d). Parallel imaging reconstruction was conducted with the autocalibration of a GRAPPA reconstruction kernel [11, 15].

At the same time, the navigator echoes following the excitation pulse were used for correcting not only the misalignment between alternating echoes along readout direction but also the intersegment amplitude discontinuities along phase-encoding direction [5]. A varying timing gap, namely, echo time shifting, was also inserted prior to data acquisition for preventing phase discontinuities of intersegment [16, 17].

### 2.2. Data Acquisition

For investigating the effect of parallel imaging on cmsEPI, we obtained images with different segments and acceleration factors, that is, 8 segments with *R* = 1, 2, and 4 or 6 segments with *R* = 1, 2, and 3. The acquisitions were performed three times on different days for reproducibility. The data with *R* = 8 in 8 segments and *R* = 6 in 6 segments were excluded, because the image reconstruction failed. Each dataset consisted of 50 volumes with which the temporal SNR (tSNR) was calculated. This experiment was performed with a spherical phantom filled with water.

Functional in vivo experiments consisted of two protocols: one was performed without any stimulus in order to analyze tSNR with the acquired 50 volumes during a resting state and the other was performed with visual stimulus, in which a flickering checker board of 8 Hz was utilized. A dummy period of 18 seconds was given prior to the initial session. A stimulus session of 18 seconds and a resting session of 18 seconds were repeated 4 times. Hence, each functional experiment was conducted for 162 seconds and 54 volumes were acquired. In functional in vivo experiment, only two conditions of 6 segments with *R* = 1 and *R* = 3 were acquired for comparison. The functional data were preprocessed and analyzed with SPM8 (The Wellcome Department of Imaging Neuroscience, London, UK).

All imaging was performed in 7T MRI (MAGNETOM, SIEMENS, Erlangen). Data acquisition parameters were as follows: field of view (FOV) 220 × 220 mm^2^, in-plane resolution 1.0 × 1.0 mm^2^, partial Fourier factor 6/8, slice thickness 1.0 mm, TE 30 ms, TR 3000 ms, 5 slices, and 3.0 mm interslice gap. Note that TR 3000 ms means the time interval between subsequent volumes. The actual TR between segments was about 55 ms per segment per slice. For the acceleration factor *R* = 3, only 2 segments were acquired with an additional temporal gap of 220 ms between slices, which corresponds to the duration of 4 segments.

### 2.3. Image Reconstruction

Based on the reference data, the images were reconstructed by using multicolumn multiline interpolation (MCMLI) with a kernel of 5 × 4  (*k*_*x*_ × *k*_*y*_), resulting in nonaliased images. Both nearest acquired line (*k*_*y*_) and column (*k*_*x*_) neighboring points were interpolated to reconstruct each missing data in the k-spaces from multiple channels [15].

To compensate for the error of intersegment, the acquired navigators were used to determine the amplitude gains of segments, in which each navigator's energy, that is, sum of square of navigator, was calculated. Then, the intersegment amplitude discontinuity was corrected by the gains. The accelerated data were recovered by GRAPPA algorithm implemented on MATLAB program (The MathWorks, Inc., Natick, Massachusetts, USA).

### 2.4. Data Analysis

Datasets for tSNR analysis were handled with a voxel-based analysis by the following equation [18, 19]:

(2) $$\begin{array}{c} tSNR\left(r\right)=\frac{{\mathrm{mean}}_{k=1\cdots K}\left(S_{N}\left(r,k\right)\right)}{{\mathrm{stdev}}_{k=1\cdots K}\left(S_{N}\left(r,k\right)\right)}, \end{array}$$

where *S*_*N*_, *r*, and *K* denoted a noised (measured) signal, a voxel position, and the number of measurements.

For in vivo dataset, region-of-interest (ROI) was determined within the gray matters chosen by threshold of the magnitude of a mean image. The threshold was selected to determine a midrange between representative intensities of CSF and white matter, which played a role in making a mask. The ROI was divided to three regions along a phase-encoding direction in order to evaluate the nonuniform loss in tSNR, and the mean and the standard deviation of voxels of each ROI or all the ROIs were evaluated.

## 3. Results

**Figure 2:**
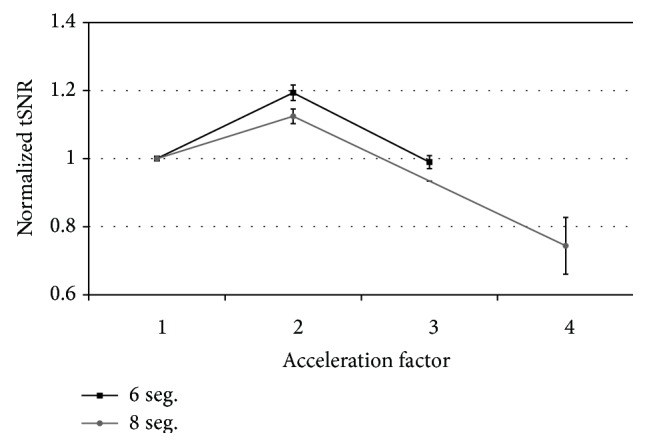
Comparison of temporal SNR at different conditions in phantom test. All of data were normalized to *R* = 1.

Figure 2 showed the normalized tSNRs at different acceleration factors of cmsEPI in a phantom test. Comparing with *R* = 1, the mean tSNR in *R* = 2 increased by 18% and 12% in 6 and 8 segments, respectively. After that, with the acceleration factors of 3 and 4, the tSNR was decreased as acceleration factor increased. The mean tSNR with 6 segments and *R* = 3 was similar to that of *R* = 1.

**Figure 3:**
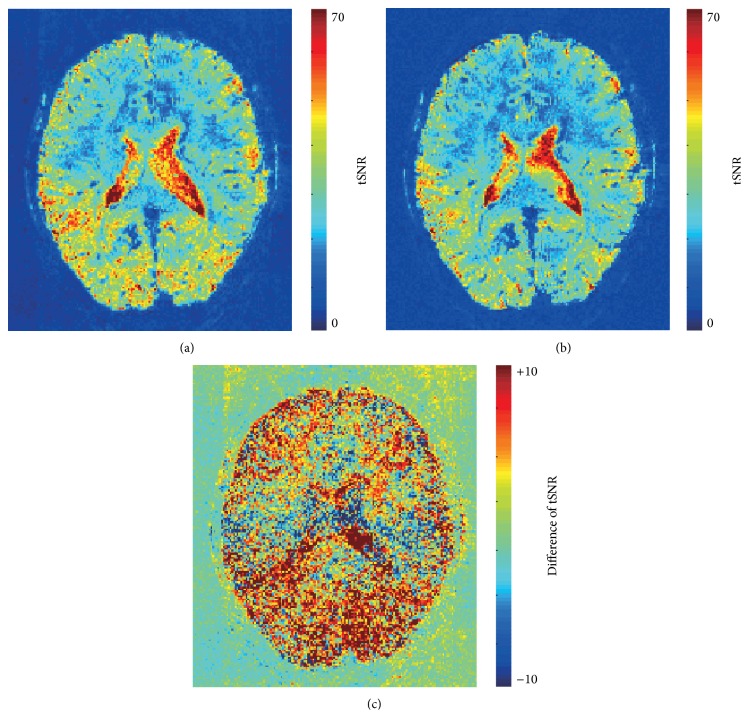
tSNR maps with/without parallel acquisition. (a) tSNR map with parallel acquisition. (b) tSNR map without parallel acquisition. (c) Difference of temporal SNR. In (c), the difference map was derived by subtraction of both maps (tSNR_*R*=3_  −  tSNR_*R*=1_).

In in vivo experiment, Figures 3(a) and 3(b) showed the tSNR maps of cmsEPI with *R* = 3 and *R* = 1, respectively. Basically, voxels including CSF tended to have a relatively high tSNR and voxels around brain ventricle had the highest tSNR. The tSNR difference between reduction factors was plotted in Figure 3(c). The most brain area of cmsEPI with *R* = 3 had a higher tSNR than that with *R* = 1 in in vivo experiment, although only the small portion of the image, especially at the midline of an image, had a decreased tSNR. For quantitatively evaluating the gain in tSNR, a mean and a standard deviation of voxels at ROIs were calculated from the tSNR difference map, and the result was presented in Table 1. The tSNR of each ROI of cmsEPI with *R* = 3 was equal to or larger than *R* = 1, and an average gain of the tSNR was about 8.2% up to 14.2%.

**Figure 4:**
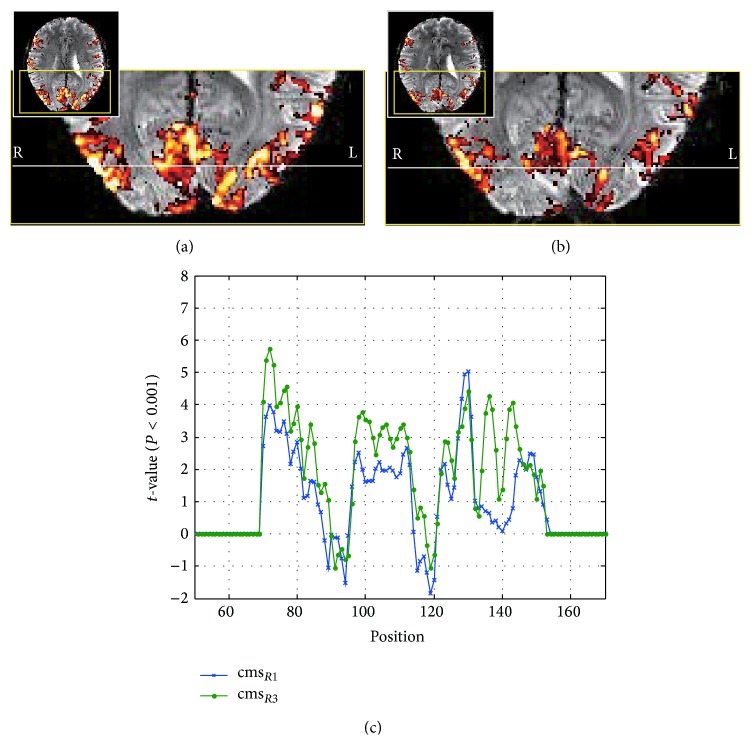
fMRI activation maps with/without parallel acquisition. (a) fMRI activation map with parallel acquisition. (b) fMRI activation map without parallel acquisition. (c) Comparison of *t*-values with/without parallel acquisition, in which *t*-values came from the white lines of (a) and (b). The improved tSNR led to entirely increased *t*-value (statistical power) to functional responses.

In functional experiments, the visual stimulus was utilized in order to observe the activations on the primary visual area of the brain. As a result, Figures 4(a) and 4(b) showed the activation maps obtained by cmsEPI with *R* = 3 and *R* = 1, respectively. To compare the two statistical values, they were displayed in the same range of *t*-value. The result of cmsEPI with *R* = 3 had larger activated areas and higher *t*-values than that of *R* = 1.  Figure 4(c) showed activation profiles in white lines on Figures 4(a) and 4(b), which showed similar patterns to each other. The profile of *R* = 3, however, provided better statistical power for activation than that of *R* = 1.

**Figure 5:**
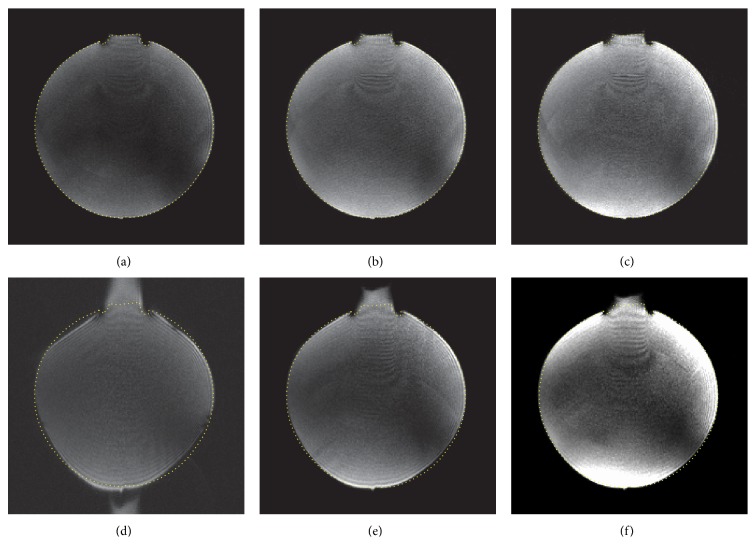
Comparison with single-shot EPI. Images were acquired by cmsEPI with 6 segments and (a, b, and c) *R* = 1, 2, and 3 and single-shot EPI with (d, e, and f) *R* = 1, 2, and 3, respectively. The yellow dotted line was drawn from the outline of (a).

Figure 5 showed the distortion comparison between cmsEPI and ssEPI. Images in cmsEPI had almost the same degree of distortion, regardless of acceleration factors, while ssEPI showed explicit geometric distortion.

## 4. Discussion

In this study, we demonstrated that cmsEPI combined with GRAPPA reconstruction provided increased tSNR compared to cmsEPI without acceleration. The image quality of the accelerated image such as signal deformation and geometrical distortion was preserved similarly to or better than the nonaccelerated image. The functional experiment to prove the functional effectiveness showed the increased functional sensitivity, in which the activated area was much broader and *t*-values were higher than in the nonaccelerated cmsEPI.

According to the previous study [20], better tSNR resulted from improved static SNR within some boundaries. Similarly, the static SNR mainly improved by the modified VFA in the parallel imaging acquisition could lead to increased tSNR. With employing acceleration acquisition, VFA were increased, leading to gain of a magnitude of transverse magnetization for each segment. When a longitudinal magnetization of *M*_0_ was given, the transverse magnetization of $M_{0}/\sqrt{n/R}$ will be applied to each segment of cmsEPI by the modified VFA. For instance, 6 segments with *R* = 1 or *R* = 3, would lead to the applied transverse magnetization of $0.4\cdot M_{0}\;(\approx M_{0}/\sqrt{6})$ or $0.7\cdot M_{0}\;(\approx M_{0}/\sqrt{6/3})$, respectively. Hence, the modified VFA by parallel imaging acquisition could produce higher strength of signal.

It should be noted that the improved tSNR seems to mitigate the disadvantage of parallel imaging reconstruction such as noise enhancement. Typically, parallel imaging such as GRAPPA resulted in a loss in tSNR by a factor of $g\sqrt{R}$, where *g* denoted nonuniform loss in tSNR based on coil geometry. With considering the transverse magnetization and the effect of parallel imaging, the tSNRs can be estimated as follows:

(3) tSNRnon∝M0n2=M02n,tSNRacc∝M0n/R2·1gR=Rg·M02n.

In (3), the superscripts of “non” and “acc” denoted a nonaccelerated and an accelerated cmsEPI, respectively. According to the equations, the tSNR of the accelerated cmsEPI entirely increased by a square root of a reduction factor but still could be decreased locally and nonuniformly by *g*-factor. Here, the variable of $\sqrt{R}$ at the equation led to improvement of the tSNR, when implemented with modified VFA described in (1). It was a contrast from the original VFA consistently decreasing the tSNR as shown in the supplementary figure, in Supplementary Material available online at http://dx.doi.org/10.1155/2015/394213, where the original VFA was in a condition without taking account of the acceleration factor of *R* in (1). Therefore, the modified VFA could essentially increase the tSNR in accelerated acquisitions.

In functional study, acceleration factor *R* = 3 was selected instead of *R* = 2, although *R* = 2 provided higher tSNR than *R* = 3. Since tSNR of *R* = 3 was similar to *R* = 1 in phantom test, the physiological effect of *R* = 3 could be investigated and compared with *R* = 1 in vivo test. And the acceleration factor of *R* = 3 or 4 has been used in most of functional experiments as well as *R* = 2 in ssEPI. In comparison of *R* = 1 and *R* = 3, tSNR difference between phantom and in vivo tests was observed due to the possible existence of physiological noise. It showed that images in *R* = 3 were less affected by physiological noise due to the shorter acquisition time so that tSNR in *R* = 3 might be better than expected in in vivo test.

It was not performed to directly compare the tSNR of the proposed method with the tSNR of ssEPI. The tSNR in ssEPI has been known to decrease with the acceleration factor, which is typically proportional to $M_{0}^{2}/g\sqrt{R}$. However, the accelerated acquisition in ssEPI could additionally provide the shorter TE due to the reduced echo train length. The reduced TE would have more influence on tSNR than the acceleration factor and the *g*-factor. In practical cases, possibly minimal TEs of ssEPI having the in-plain resolution of 1.0 mm^2^ were 68 ms and 29 ms in *R* = 1 and *R* = 3, respectively. Considering a voxel with T_2_^*∗*^ of 60 ms, the signal intensity in TE of 29 ms would apparently be about 1.9 times higher than in TE of 68 ms. Thus, since the tSNR was determined by imaging parameters, the direct comparison of the tSNR would not be necessary. As the same TE was given, the proposed method would provide lower tSNR than ssEPI.

Artifacts on the image could occur by intersegment modulations arising from VFA, which would occur in both magnitude and phase. The magnitude modulation would be caused by a nonideal shape in a slice-selective RF profile and different sensitivities of a RF coil to various flip angles, but it could be almost compensated by the comparison of navigators of segments. The intersegment phase modulation would be also caused by B1 field differences of various flip angles, that is, signal phase difference between practical excitation RF pulses with 45° and 90°. In contrast to the magnitude modulation described above, the level of the artifact by the phase modulation could be changed in the region where B1 significantly deviates from the nominal one. Further study will be needed for handling these artifacts.

The proposed method has the similar image contrast with conventional ssEPI, because cmsEPI with parallel imaging can preserve the image contrast given by the same TR and TE as ssEPI. The image contrast of a typical msEPI, however, includes the different T1 recovery effect as well as the T_2_^*∗*^ relaxation effect due to a time interval between excitation RF pulses, compared with ssEPI.

In addition, the proposed method with the shorter echo time and the echo train length could function as an alternative and controllable geometrical distortion correction technique, leading to reduction in the geometrical distortion inherently and preventing of the signal loss by field inhomogeneity. Though the postprocessing distortion correction techniques such as PSF-mapping can produce a distortion-corrected image similar to the distortion-free gradient-recalled echo image (GRE) [21], the information loss by a fast T_2_^*∗*^ decay is hard to be recovered completely. Therefore, the proposed method is possible to be easily implemented with other postprocessing correction techniques.

The proposed method also has the potential capability of further improving the imaging coverage. The future extension of the proposed method for imaging coverage can be achieved by multiband (MB) or simultaneous multislice (SMS) techniques based on 2D imaging [22]. However, direct 3D approach using multiple excitations should be carefully applied to the proposed method, because minimum intersegment can be conflicted by multiple excitations with constant interval or too many segments can cause the decrease in static SNR of an image.

## 5. Conclusions

This study proposed an advanced technique of cmsEPI for functional study. We demonstrated that the combination of cmsEPI with parallel imaging acquisition could provide a synergic effect to improve functional sensitivity.

## Supplementary Material

The supplementary figure showed the effect of modified variable flip angle (mVFA) on the temporal SNR. The temporal SNRs in mVFA were substantially increased as compared with original variable flip angle (oVFA). Note that all the data were normalized to R=1.

## Figures and Tables

**Table 1:** Comparison of averaged tSNRs on ROIs selected from the gray matter compartment.

	*R* = 1	*R* = 3	Gain (%)
ROI 1	33.7 ± 7.6	38.5 ± 9.0	+14.2
ROI 2	34.1 ± 7.9	34.3 ± 7.9	+0.6
ROI 3	30.4 ± 6.9	32.2 ± 7.5	+6.0
Whole ROIs	33.1 ± 7.7	35.8 ± 8.7	+8.2
