# Supplementary material for: Fast Imaging Technique for fMRI: Consecutive Multishot Echo Planar Imaging Accelerated with GRAPPA Technique
Source: Biomed Res Int. 2015 Aug 27;2015:394213. doi: 10.1155/2015/394213 (PMC4564598; doi:10.1155/2015/394213)

Supplementary Figure. Effect of modified variable flip angle (mVFA) on temporal SNR. The temporal SNRs were substantially increased by mVFA with accelerated acquisitions relative to original VFA (oVFA). Note that all the data were normalized to R=1.

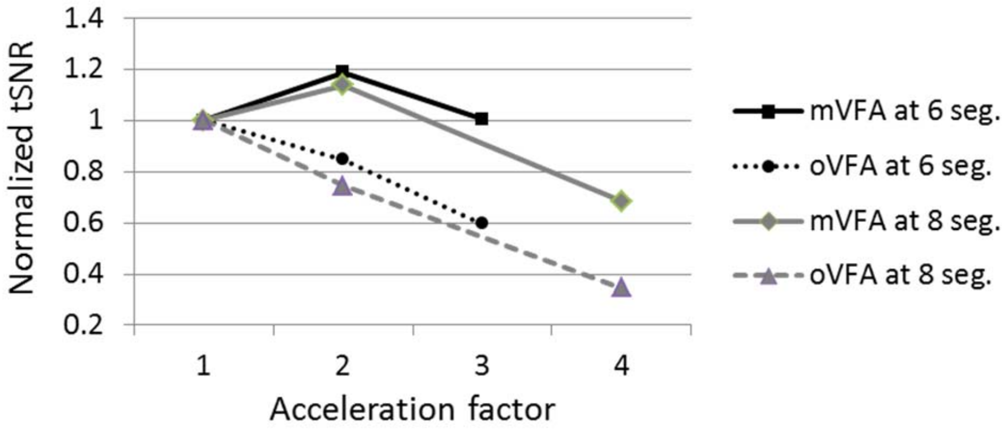

Supplement: Supplementary file 1 — The supplementary figure showed the effect of modified variable flip angle (mVFA) on the temporal SNR. The temporal SNRs in mVFA were substantially increased as compared with original variable flip angle (oVFA). Note that all the data were normalized to R=1. [file 394213.f1.pdf]
